# Complications of silicone oil in vitreoretinal surgery: a narrative review of clinical and experimental evidence

**DOI:** 10.1186/s40942-026-00847-w

**Published:** 2026-04-02

**Authors:** Rosa Lomelino Pinheiro, Filipe Henriques, Mun Faria, Elisa Julião Campos, Carlos Marques-Neves

**Affiliations:** 1 Ophthalmology Department, Local Health Unit of Santa Maria, Avenida Egas Moniz, Lisboa, 1649-028 Portugal; 2 Clínica Universitária de Oftalmologia da Universidade de Lisboa, Lisbon, Portugal; 3 Centro de Responsabilidade Integrado de Oftalmologia, Coimbra Local Health Unit, Coimbra, Portugal; 4 CEReS, Department of Chemical Engineering, University of Coimbra, Coimbra, Portugal; 5 Center for Neuroscience and Cell Biology, University of Coimbra, Coimbra, Portugal; 6 Center for Innovative Biomedicine and Biotechnology, University of Coimbra, Coimbra, Portugal

**Keywords:** Silicone oil, Silicone oil-related visual loss, Emulsification, Glaucoma, Sub-silicone oil fluid

## Abstract

Our purpose was to review the existing literature on the clinical and immunopathologic adverse effects of silicone oil (SO). A review was performed of studies published from 1993 to October 2025 in PubMed/MEDLINE, Web of Science, ClinicalTrials.gov, and Cochrane Library. Heavy SO agents were excluded. For silicone oil-related visual loss (SORVL), only rhegmatogenous retinal detachment studies reporting macular status were included. The review is organized into subthemes, including SORVL, emulsification, SO-related corneal and lens changes, retinal layer abnormalities, vascular changes, glaucoma, electrolyte balance, globe deposits, and optic neuropathy. Currently, there is no consensus explanation for SORVL, either due to the different surgical methodologies, the complexity of cases, or the lack of routine multimodal testing. SORVL was frequently associated with inner macular thinning, papillomacular bundle damage, and reduced macular vessel density. Emulsification and globe deposits have been described as a possible cause for the irreversible nature of some adverse effects. Longer SO duration was frequently, but not always, related to structural ocular changes. Finally, cytokine and electrolyte profiles provide evidence of inflammatory and potentially toxic mechanisms. In this review, we compiled the most recent evidence on the structural and functional complications associated with SO (such as macular thinning, SORVL, and optic neuropathy). We then summarized the pathophysiological mechanisms that may lead to these complications, including inflammation, phototoxicity, emulsification, and globe deposits. Standardized definitions (particularly for SORVL and SO-related ocular hypertension), multimodal monitoring protocols, and consensus on safe tamponade duration are still lacking.

**Supplementary Information:**

The online version contains supplementary material available at 10.1186/s40942-026-00847-w.

## Introduction

Silicone oil (SO) is a vitreous substitute widely used in vitreoretinal surgery, particularly for the management of complex rhegmatogenous retinal detachments (RRD), including those associated with giant retinal tears, proliferative vitreoretinopathy (PVR), as well as in selected non-RRD indications such as proliferative diabetic retinopathy (PDR) and endophthalmitis.

SO is a synthetic polymer composed mainly of polydimethylsiloxane, characterized by a backbone of alternating silicon and oxygen atoms with methyl side groups [1, 2]. This molecular configuration confers high chemical stability, optical transparency, and low surface tension, properties that allow it to replace the vitreous while maintaining retinal reattachment [3–5]. However, other physicochemical characteristics, particularly viscosity, interfacial behavior, and the presence of low molecular weight components (LMWC), also influence the interaction of SO with ocular tissues and may contribute to the development of postoperative complications [1]. While SO’s ability to stabilize the retina is well-documented [6], the long-term complications associated with SO remain a topic of ongoing debate [7, 8].

Several hypotheses have been proposed to explain SO-related complications, including ocular hypertension (OHT), the infiltration of emulsified SO into ocular tissues, direct retinal toxicity, inflammatory cytokine involvement, and phototoxicity, among others [9–11]. However, despite extensive research, a clear consensus on the pathophysiology of SO-related complications, particularly SO-related visual loss (SORVL), is lacking [12–14].

Whereas previous reviews primarily categorized SO-related complications according to the ocular structure involved,^11,15^ the present review focuses on the pathophysiological mechanisms leading to those adverse effects. By structuring complications according to mechanisms such as emulsification-related toxicity, mechanical effects, and inflammatory or physiological alterations, we provide a more integrative understanding of SO-associated morbidity. For each mechanistic category, we summarize the available evidence, discuss proposed mechanisms of damage, and highlight current gaps in knowledge that warrant further investigation. Furthermore, this review deliberately focuses on the use of conventional SO in RRD surgery (while also distinguishing macula-on from macula-off RRD, whenever possible), excluding heavy SO and other clinical indications such as PDR or endophthalmitis, in order to minimize confounding factors and allow a more precise assessment of SO-specific complications, in contrast to previous articles on the topic [2, 11].

This narrative review critically evaluates the current literature on SO complications, analyzing its interactions with ocular structures from mechanical, immunopathological, and vascular perspectives. Additionally, we highlight key knowledge gaps and propose potential clinical guidelines for managing SO complications (Table 1).

**Table 1:** Silicone oil in retinal surgery: summary of complications, knowledge gaps, and research directions

Complication	Clinical and scientific findings	Gaps in knowledge	Clinical or research recommendations
Silicone oil-related visual loss	• Vision loss in SORVL is often confirmed using VA and microperimetry testing • Not all studies found an association with OHT, and visual defects are not glaucoma-like • Electrophysiology tests consistently show macular dysfunction • May develop during SO tamponade but can remain undetected until ROSO • Incidence is highly variable and likely underreported • SO may dissolve fat-soluble protective pigments, such as lutein and zeaxanthin, increasing vulnerability to phototoxicity	• Risk stratification • Relative contributions of the mechanisms proposed, including mechanical stress, vascular compromise, and direct toxicity • Impact of surgical technique, SO duration, and viscosity • Timing of symptoms (that are evident only months after surgery) challenges the idea of acute light toxicity	• Differentiating patients with macula-on and -off RRD in SORVL studies • Standardizing diagnostic workup by: i. Performing microperimetry and ERG during and after ROSO ii. Measuring VA before RRD surgery, before and after ROSO • Investigating SO’s potential to dissolve macular pigments (lutein, zeaxanthin) and impact foveal protection
Emulsification	• Surfactants, the remaining vitreous, and kinetic energy favour emulsification • LMWC are the most abundant impurities in SO and increase emulsification • The clinical impact of SO viscosity observed in vitro may not translate to patient outcomes	• Role of LMWC in clinical toxicity, namely, in vivo significance, dose-response, and safe thresholds • Systematic use of grading systems, namely a recent one including macular and optic disc OCT	• Assessing the influence of surgical manoeuvres such as encircling bands, avoiding turbulence and complete SO fill • Measuring safely and reproducibly emulsification of SO within the globe • Studying the physical properties of SO, besides viscosity and emulsification
SO-related corneal and lens changes	• SO-associated keratopathy includes endothelial loss, pleomorphism, stromal deposits, and edema, with incidence ranging from 2–30% • Mechanisms involve mechanical obstruction by emulsified droplets, toxic/apoptotic endothelial effects, and subclinical damage detected by confocal microscopy • Risk increases with longer SO retention, anterior migration, and OHT; evidence on viscosity-related differences remains inconsistent • Cataract progression is highly prevalent after SO tamponade	• Relative contribution of surgical factors (e.g., aphakia, cataract surgery) versus SO migration to endothelial loss is unclear • Clinical impact of nanosized emulsified droplets and the role of SO viscosity remain uncertain • Lack of standardized early diagnostic markers of endothelial toxicity	• Minimize anterior migration (e.g., maintain lens–iris diaphragm, consider aphakic retention sutures) • Use specular/confocal microscopy for early detection of endothelial changes; consider earlier SO removal • Consider combined phacoemulsification with intraocular lens implantation at the time of SO tamponade
Retinal layers	• Thinning of the inner retinal layers is frequent in patients with and without SORVL • Hypotheses for the structural alterations of the retinal layers are: i. degeneration of Müller cells ii. infiltration of emulsified SO or of its LMWC, after dissolution of cellular membranes iii. mechanical stress to the fovea iv. secondary to optic neuropathy v. secondary to changes to the choroidal blood flow	• If changes (especially in inner and outer retinal layers) are due to SO itself or the underlying retinal detachment, especially in macula-off cases • Standardized measurement techniques, scan quality with *SO in situ*, and follow-up durations to allow direct comparisons	• Clearly dividing eyes into macula-on and macula-off RRD • Specifying whether OCT measurements were obtained with SO in situ or post-removal • Clarifying the comparator (e.g., fellow eye, gas-treated eye, longitudinal intra-eye changes) • Correlating OCT findings with visual acuity and other functional tests
Vascular changes	• Reduced vessel density in the superficial capillary plexus in SO-filled eyes, regardless of macular status • Retinal arteriole narrowing after > 9 months of SO tamponade, likely due to neuroretinal toxicity or oxidative stress • Longer arteriovenous passage time, reduced retinal blood flow, especially compared to gas	• If retinal and choroidal circulation fully recover after SO removal, especially in the long term • If increased choriocapillaris flow caused by inflammation is driven by SO, endolaser, cryopexy, or both • If blood flow parameters (e.g., OCT-A metrics) are more sensitive than structural thickness	• Performing OCT-A before ROSO and during the postoperative period • Standardizing the measurement of choroidal and choriocapillaris blood flow • Routinely measuring of choroidal thickness
Ocular hypertension and silicone oil-related glaucoma	• Ocular hypertension is more common after vitrectomy with than without SO • Reported incidence varies widely (11–56%), reflecting study heterogeneity • Emulsification and LMWC contribute to trabecular damage and inflammation • Central vision loss is more common than glaucoma-like defects on VF	• Preoperative predictors of SO-related glaucoma • Treatment thresholds and diagnostic criteria (e.g., role of IOP spikes, optic disc changes) • Link between central vision loss and IOP spikes	• Clarifying the definition of OHT and glaucoma • Performing VF after ROSO • Evaluating IOP spikes, using 24-hour tonometry
Electrolyte balance and cytokines during SO	• SO absorbs lipophilic substances, such as cholesterol and lipophilic acids • Cytokine and metabolic profiles suggest an inflammatory microenvironment beneath SO, particularly in eyes with proliferative vitreoretinopathy or PDR	• Studies specifically addressing RRD • Comparison of SSOF, vitreous, and aqueous from the same eyes or disease groups • If observed reductions in proinflammatory cytokines are protective or merely sequestration phenomena	• Establishing electrolyte and cytokine “normal ranges” for SSOF • Correlating biochemical abnormalities with clinical endpoints (e.g., vision loss, retinal thinning) • Standardizing sampling techniques (e.g., SSOF *versus* vitreous) to improve comparability
Globe deposits	• SO can migrate into various ocular tissues and provoke inflammatory or toxic changes • Histological and imaging evidence supports the presence of SO within the retina, optic nerve, anterior chamber, and subretinal space • These changes can occur regardless of viscosity or duration of tamponade	• Irreversible visual loss may be linked to retained intraretinal SO or optic nerve infiltration, but causal evidence remains limited in humans • Standardized data on the cytotoxic effects of LMWC in vitro and in vivo	• Understanding how SO damages the outer retinal layers • Clarity on whether intraretinal SO migration is promoted by surgical techniques (e.g., ILM peeling, retinectomies) • Understanding of the clinical relevance of SO migration to the optic nerve
Optic neuropathy	• SO may migrate into the optic nerve and exert direct toxic effects or inflammatory damage, with or without elevated IOP • Histology shows SO droplets within the optic nerve surrounded by macrophages (“pseudo-Schnabel’s cavernous degeneration”) • Retrolaminar SO migration has been documented in enucleated eyes, regardless of SO viscosity • Most affected eyes had secondary glaucoma, though optic disc cupping was uncommon	• Limited understanding of the mechanisms driving SO entry into the optic nerve (congenital disc anomalies, OHT-related infiltration, phagocytosed droplets) • Unclear clinical significance of SO migration beyond the globe (e.g., into cerebral ventricles) • Lack of consistent criteria to distinguish SO-related optic neuropathy from glaucomatous or ischemic damage	• Monitor patients with prolonged SO tamponade or OHT for early signs of optic nerve dysfunction • Standardize diagnostic criteria and imaging markers (e.g., OCT/RNFL profiling, neuroimaging) to identify SO-related neuropathy • Investigate pathways of SO migration and clarify the contribution of OHT, congenital anomalies, and phagocytic transport

ERG: electroretinography; IOP: intraocular pressure; ILM: internal limiting membrane; LMWC: low molecular-weight components; OCT: optical coherence tomography; OCT-A: optical coherence tomography angiography; OHT: ocular hypertension; PDR: proliferative diabetic retinopathy; ROSO: removal of silicone oil; RPE: retinal pigment epithelium; RRD: rhegmatogenous retinal detachment; SO: silicone oil; SORVL: silicone oil-related visual loss; SSOF: sub-silicone oil fluid; VA: visual acuity; VF: visual fields

## Materials and methods

### Search strategy

A comprehensive and international literature search was performed to gather relevant studies on SO complications from 1994 to October 2025. The databases utilized for this literature search included PubMed/MEDLINE, Web of Science, ClinicalTrials.gov, and Cochrane Library. The following Medical Subject Headings (MeSH) search terms were used: “silicone oil” AND “emulsification”, “vision loss”, “complications”, “cornea”, “keratopathy”, “cataract”, “epiretinal membrane”, “cystoid macular edema”, “optic nerve”, “ocular hypertension”, “glaucoma”, “emulsification”, “subsilicone oil fluid”, “retro-oil fluid”, and “cytokines”. Language was restricted to English, Portuguese, Spanish, and Italian. References of retrieved articles and recent reviews were hand-searched for additional publications. Given that this is a review of previously published studies, no new ethical approval was required. This review was not registered, and no review protocol was prepared.

### Selection criteria

Two authors (RLP and EJC) independently screened titles and abstracts, with disagreements resolved through open discussion with the other authors. Full texts were then assessed for eligibility based on the predefined inclusion criteria and methodology. Studies using heavy SO were excluded, as these agents differ substantially from conventional SO in terms of physical properties and complication profiles.

We preferentially cited studies involving RRD, particularly when addressing the incidence and prevalence of clinical complications, since other pathologies such as PDR and endophthalmitis carry a worse visual and anatomical prognosis, require more complex surgical maneuvers and longer operative times, and have a strong proinflammatory component. Accordingly, RRD studies were prioritized for clinical outcome interpretation, whereas non-RRD studies were included selectively to support mechanistic insights.

### Data extraction

Two authors independently extracted data and disagreements were resolved by discussion. For each eligible report we collected: study characteristics, population and baseline factors, viscosity of SO, duration of tamponade, and follow-up length. Outcomes were complications associated with SO. When data were missing or unclear, no imputation was performed and the information was reported as not available. Results were summarized using a structured table and illustrative figures, organized by pathophysiological mechanisms.

### Main text

A total of 197 results were obtained from the literature search. In total, 130 studies were identified and considered pertinent for this review. Key findings were categorised into the following themes: (A) SORVL, (B) Emulsification of SO, (C) SO-related corneal and lens changes, (D) Retinal layers, (E) Vascular changes associated with SO use, (F) Ocular hypertension and SO-related glaucoma, (G) Electrolyte balance and cytokines during SO, (H) Globe deposits, and (I) Optic neuropathy.

### Limitations

This review has several limitations. First, the available evidence is predominantly derived from retrospective studies and case series, which inherently limits the strength of the conclusions. Second, there is substantial heterogeneity across studies, including differences in study design, surgical indications, SO tamponade duration, viscosity, and outcome definitions, which complicates direct comparisons. Third, the lack of standardized outcome measures across studies further limits the ability to synthesize findings consistently. Additionally, this body of literature is subject to potential sources of bias, including selection bias, reporting bias, and unmeasured confounding. These factors, combined with the observational nature of most included studies, restrict the ability to draw causal inferences or perform robust comparative analyses. Finally, although this review aimed to focus primarily on RRD to reduce confounding and improve internal validity, studies involving other indications (such as PDR, PVR, and endophthalmitis) were included in selected sections, particularly in mechanistic discussions. This approach may introduce additional heterogeneity and should be considered when interpreting the overall findings.

## Silicone oil complications

### Silicone oil-related visual loss

#### Definition and incidence

SORVL is characterized by a significant, often irreversible loss of central vision (> 2 Snellen lines) which cannot be attributed to the underlying retinal pathology or any other identifiable cause [10]. Reported incidence rates of SORVL range widely – from 3.3% to 50% – reflecting potential underreporting and the absence of standardized diagnostic criteria [12, 13, 16].

#### Functional and structural changes

**Fig. 1:**
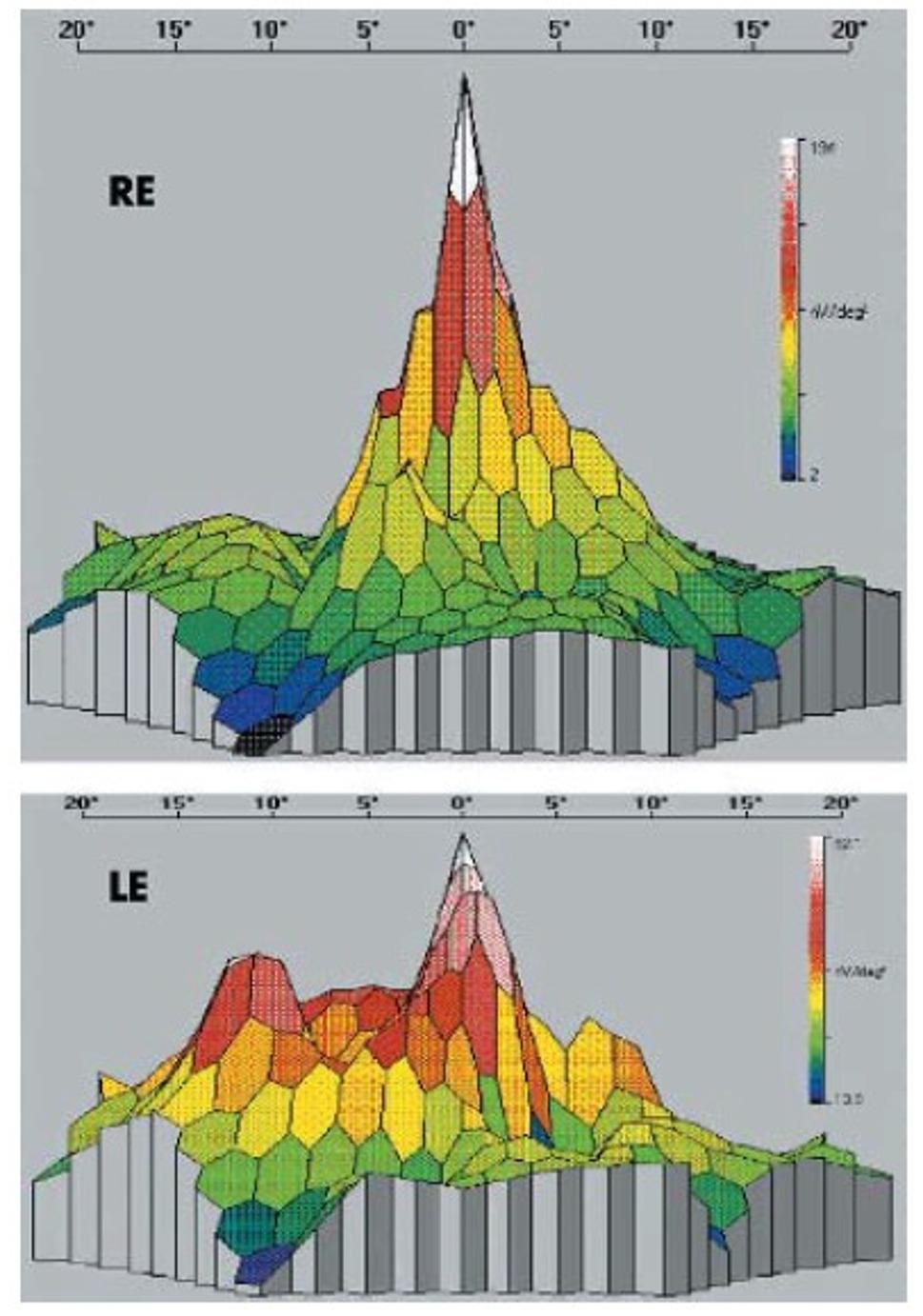
Multifocal ERG responses comparing affected right eye with unaffected left eye: case 1 [15]. Reprinted with permission from Oliveira RA, Magalhaes Junior O, Rossi JPDS, et al. Complications of silicone oil as vitreous tamponade in pars plana vitrectomy: a mini review, Copyright 2025, Curr Eye Res

In patients who develop SORVL following RRD repair, electrophysiologic testing frequently reveals macular dysfunction that exceeds expectations based solely on the detachment itself (Fig. 1) [13, 17–21]. Visual evoked potentials are generally normal [18], though some studies have described optic nerve involvement [13, 22]. Visual field testing commonly shows central scotomas, while glaucomatous-type defects are typically absent [21, 23]. Microperimetry studies demonstrate decreased foveal sensitivity in patients with SORVL [22, 24] and in both macula-on and -off RRD eyes treated with SO when compared with gas [23].

#### SORVL and intraocular pressure

The relationship between SORVL and intraocular pressure (IOP) remains debated. While several studies report no significant differences in IOP between patients with and without SORVL [12, 17, 20, 25–28], others have observed higher mean IOP in affected patients [20, 24, 26]. In the Pan American Collaborative Retina Study, elevated IOP has been identified as a potential risk factor for SORVL, particularly after removal of SO (ROSO) [14]. In one study, elevated IOP was the strongest predictor of vision loss, with pressure spikes consistently preceding visual decline [16]. The pattern of vision loss was predominantly central, not peripheral, and was attributed to selective retinal nerve fiber layer (RNFL) damage in the papillomacular bundle [16]. On one hand, this may result from trans-synaptic degeneration triggered by inner retinal emulsified SO droplets, mimicking the microcystic changes seen in other optic neuropathies (e.g., multiple sclerosis, neuromyelitis optica, hereditary optic atrophy) [16, 26]. It is also plausible that transient IOP spikes contribute to mechanical stress on RNFL – already more vulnerable due to altered biomechanics following vitrectomy and SO [16]. Finally, such pressure fluctuations may reduce ocular perfusion pressure, potentially impairing axoplasmic transport within the papillomacular bundle [16].

#### SORLV and OCT findings

**Fig. 2:**
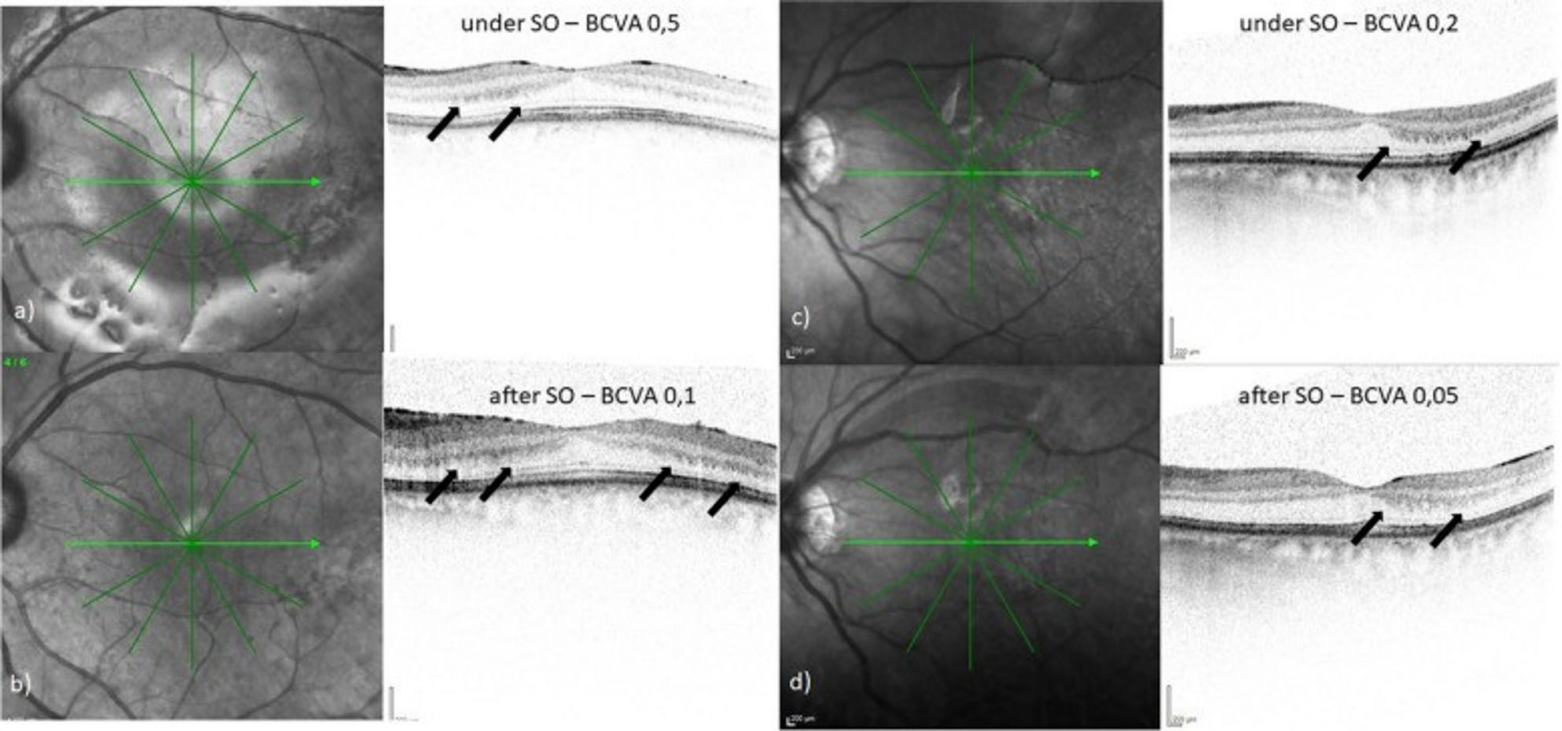
Follow-up OCT images of 2 eyes with unexplained visual loss (UVL) under SO tamponade, showing alterations at the level of the outer plexiform layer (OPL) corresponding to higher reflectivity of Henle’s fibre layer. Left eye of a 63-year-old woman with UVL after 4 months, duration of SO tamponade of 5 months and OPL changes (→) during (**a**) and after (**b**) SO tamponade. Left eye of a 50-year-old man with UVL after 4,5 months, duration of SO tamponade of 4 months and OPL alterations (→) during (**c**) und after (**d**) SO tamponade. [13] Reprinted with permission from Ref [Barth T, Helbig H, Maerker D, et al. Unexplained visual loss after primary pars-plana-vitrectomy with silicone oil tamponade in fovea-sparing retinal detachment, Copyright 2023, BMC Ophthalmol]. https://creativecommons.org/licenses/by/4.0/#ref-appropriate-credit

In eyes with SORVL, several studies noted preferential thinning of the inner retinal layers, especially within the papillomacular bundle [26, 27]. Again, this region, due to its high metabolic activity, may be particularly vulnerable to pressure, vascular, or mechanical effects of SO [27]. Müller cell degeneration, evidenced by optical coherence tomography (OCT)-based thinning of the inner retinal layers, has been proposed as a potential pathophysiological mechanism [29]. However, one long-term study of 22 macula-on RRD eyes found no difference in central macular thickness between those with and without SORVL (Fig. 2) [12].

#### SORVL and phototoxicity

Phototoxicity has been proposed as a contributing factor in the development of SORVL, particularly in cases where electroretinogram abnormalities have been detected. A proposed mechanism involves increased retinal light exposure due to the optical properties of SO, which transmits more blue light than the natural vitreous. Shorter-wavelength blue light is known to be particularly damaging to foveal photoreceptors and retinal pigment epithelial (RPE) cells, especially since these regions are less protected by overlying ganglion cells [30, 31]. Additionally, due to its higher refractive index and the varying curvature of SO bubbles, SO can create an uneven illumination of the retina through an optical vignetting effect. This may result in transient focal increases in foveal light exposure, leading to photoreceptor damage [30, 32].

Clinical observations support this theory. In one study, 5 out of 114 eyes developed SORVL after SO removal under direct microscope illumination, compared to none of 78 eyes when light was blocked during ROSO [30]. However, the follow-up in that study was relatively short (less than 3 months), and RPE changes that would support phototoxicity were not evident on fluorescein angiography. [19] Furthermore, in a series of 12 patients with SORVL (5 of whom had visual loss while SO was still in place), visual symptoms developed months after surgery, not immediately postoperatively, casting some doubt on acute light toxicity as the sole mechanism. [19] In one case, macular pigment optical density was significantly reduced in the affected eye compared to the contralateral eye, suggesting a possible compromise in the retina’s natural photoprotective mechanisms. [33] Macular pigments like lutein and zeaxanthin protect against blue light and oxidative damage. Some authors have speculated that SO may dissolve these fat-soluble pigments, potentially lowering the threshold for phototoxic injury. [30, 33] Another proposed mechanism involves damage to mitochondrial chromophores in the retinal ganglion cell axons. In this scenario, ganglion cell damage would not be visible on fluorescein angiography. [31].

In summary, SORVL is an underrecognized complication characterized by predominantly central, often irreversible vision loss, likely driven by a multifactorial interplay of inner retinal vulnerability, pressure-related stress, and altered retinal physiology, including possible phototoxic mechanisms, rather than by a single isolated cause.

### Emulsification of silicone oil

Emulsification refers to the dispersion of fine SO droplets within another fluid, typically aqueous, due to shearing forces. This process requires a reduction in surface tension, which is facilitated by the presence of surfactants [34, 35]. These surfactants, such as serum components, fibrin, fibrinogen, and low-density lipoproteins, are more abundant in inflammatory or haemorrhagic intraocular environments, increasing the risk of emulsification [34]. Besides, the emulsified SO droplets themselves can trigger inflammatory responses, as they can be phagocytised by macrophages, microglial and RPE cells [36]. Emulsification is also influenced by the rheologic characteristics of the SO, viscoelastic solutions, proteins, lipids, ionised solutions, and impurities in the SO [5, 35, 37]. It is generally considered that emulsification is time-dependent: the longer SO remains in the eye, the more likely the emulsification is [5, 34, 38].

Assessing SO emulsification in vivo is challenging, since small droplets (< 1 μm) are not detectable by slit-lamp biomicroscopy or gonioscopy [39]. A novel grading system has been proposed that incorporates not only slit-lamp and fundus examination but also macular and optic disc OCT, where SO-related hyperreflective dots can be identified [40].

#### Mechanical and dynamic factors

Mechanical agitation, such as that due to eye movements, contributes to emulsification, as shear stresses generated during eye rotations at the SO-aqueous interface destabilize that interface [36, 39]. Patients with nystagmus have shown earlier and more severe SO emulsification, likely due to increased ocular motion [41, 42]. Using an artificial eye model, it was demonstrated that an encircling band stabilizes the SO bubble during eye movements, reducing emulsification [35, 43].

#### Surgical considerations

Residual vitreous appears to play a significant role. In vitro studies showed that SO forms smaller and more stable droplets, when in contact with vitreous compared to aqueous humour, suggesting that incomplete vitrectomy may promote emulsification [44]. Additionally, achieving a more complete fill of the vitreous cavity reduces the aqueous phase and minimizes the interface area, which lowers friction and the risk of emulsification [35, 43].

Regarding the use of SO and its interaction with other intraocular medical devices and surgical procedures, we examined the influence of scleral buckling, retinectomies, endolaser photocoagulation, heavy liquids, triamcinolone, and surgical dyes in conjunction with SO. The use of a scleral buckle appears to reduce the propensity for SO emulsification [43]. In addition, an in vitro study demonstrated that SO exposed to green argon laser irradiation showed a greater tendency to emulsify [45]. Another in vitro study recommended avoiding turbulence at the interface between SO and heavy liquids, as this may promote droplet formation [46]. Histopathological studies have further shown that the presence of SO deposits is closely associated with retinectomy sites [47]. Finally, there seems to be a cumulative or synergistic retinal toxic effect of retained surgical materials when used in combination with SO, such as triamcinolone, blue dye and perfluoro-n-octane [48]. Importantly, in vitro testing demonstrated that each compound, when assessed individually, did not induce cytotoxicity, indicating that retinal damage was primarily associated with the combined use and persistence of residual substances rather than exposure to any single agent alone [48]. In clinical studies, the use of triamcinolone has been associated with a reduced risk of ERM formation after surgical repair of RRD with SO [49, 50], whereas no protective effect has been demonstrated for cystoid macular edema (CME) [51].

#### Role of silicone oil viscosity

Lower-viscosity formulations are easier to inject and remove from the vitreous cavity, and tend to exhibit greater chemical purity, as increasing polymer chain length is associated with enhanced retention of LMWC [1, 2]. On the other hand, higher-viscosity SO (e.g., 5000 cSt) is less prone to emulsification, under laboratory conditions, due to its increased molecular weight [5, 34, 37]. Also, increasing SO viscosity increases resistance to volume displacement, in in vitro RD models [52]. However, an in vitro study showed that shear viscosity did not have a major influence on the propensity of SO to emulsify [43]. Besides, clinical studies have not consistently shown significant differences in emulsification rates between 1000-cSt and 5000-cSt SO, likely due to the overriding effect of surfactants present in vivo [5, 53, 54]. Importantly, tamponade efficacy is not influenced by viscosity, as the interfacial surface tension (rather than viscosity) determines the tamponading force [55].

Also, in a several papers analyzing the difference between low and high SO viscosity, there were no differences in the anatomical and functional outcomes and in the incidence of post-operative complications in most studies [11, 52, 56]. Some studies suggest that elevated IOP may be more pronounced with higher viscosity SO [5, 52,54], although others contradict this hypotheses [57, 58]. One in vitro study also found higher keratopathy rates with 5000-cSt SO compared with 1000-cSt SO [3, 59], while another author concluded that lower viscosity SO could lead to a higher prevalence of CME [51].

#### Impurities and toxicity

LMWC are the most abundant impurities in both liquid and emulsified SO and are strongly implicated in increasing emulsification frequency [60]. Some studies suggest that these components may also have cytotoxic effects. In vitro studies using ARPE-19 retinal pigment epithelial cells have shown similar reductions in cell viability, with both 1000-cSt and 5000-cSt SO, suggesting that viscosity itself does not mitigate cytotoxicity. [61]. While ganglion cells are in more direct contact with SO in vivo, their study is limited by technical challenges, including the lack of specific markers and frequent cellular contamination [60].

Summing up, emulsification of SO is a multifactorial process influenced by time, inflammation, oil purity, and surgical technique. It is well-established that early ROSO, complete vitrectomy, and minimizing contact with intraocular substances reduce emulsification risk. However, the clinical significance of oil viscosity differences is still debated, the exact contribution of LMWC to toxicity is not fully understood and would benefit from prospective comparative studies to clarify their clinical relevance.

### Silicone oil-related corneal and lens changes

SO-associated keratopathy encompasses a spectrum of pathologic changes, including band keratopathy, endothelial cell loss, endothelial pleomorphism and polymegathism, stromal deposits, and corneal edema. The reported incidence varies widely, from 2% to up to 30%, reflecting differences in patient selection, detection methods, and tamponade duration [3, 62].

The pathophysiology of SO-related keratopathy is multifactorial. Direct contact between emulsified oil droplets and the corneal endothelium produces a mechanical barrier that disrupts aqueous flow and interferes with endothelial nutrition. [3] When SO is in situ, it may limit stromal hydration; however, after its removal, corneal decompensation may ensue. Moreover, prolonged exposure exerts a toxic effect on endothelial cells, inducing apoptosis [62]. Even in eyes without visible oil in the anterior chamber (AC) [63], in vivo imaging has demonstrated subclinical endothelial alterations, suggesting that nanosized emulsified droplets undetectable at the slit lamp may still impair endothelial metabolism [62, 64, 65]. Confocal microscopy has revealed hyperreflective stromal deposits and morphologic endothelial abnormalities in up to 75% of eyes with SO in the AC and in approximately 40% of SO-filled eyes overall, even when slit-lamp findings were normal [64].

Several ocular and surgical factors modulate the severity of SO-induced corneal injury. Longer retention times strongly correlate with more emulsification and migration to the AC [62]. The presence of glaucoma or sustained OHT exacerbates endothelial damage through additional mechanical and metabolic stress [62]. However, in patients using pressure-lowering agents, the potential corneal side effects of these medications may also be considered [66]. Several studies report that aphakic eyes and those with SO migration into the AC are at highest risk for endothelial cell loss [3, 62]. Higher-viscosity SO show slightly lower emulsification in experimental models, but clinical data remain inconclusive; some reports even document higher keratopathy rates and higher inhibition of corneal endothelial cells proliferation in vitro with 5000-cSt SO compared with 1000-cSt SO [3, 59].

Prevention and management strategies focus on minimizing emulsification and endothelial contact. Intraoperatively, maintaining an intact lens-iris diaphragm, or, in aphakic eyes, using retention sutures, can reduce anterior migration and prevent higher endothelial cell density losses [3, 66, 67]. Postoperatively, early detection of endothelial alterations is achievable using specular or confocal microscopy, enabling timely intervention [62].

Cataract progression following SO use has been reported in up to 90% of patients when follow-up is sufficiently long [68, 69], and it is both more frequent and occurs earlier compared with gas or air tamponade [70, 71]. Several mechanisms have been proposed to explain cataract development after SO, including metaplasia of the lens epithelial cells and toxicity resulting from direct contact between SO and the posterior lens capsule, leading to alterations in lens metabolism [11, 69]. Cataract surgery in SO-filled eyes may result in variable refractive outcomes, most commonly a hyperopic shift [72]. In addition, cataract surgery after ROSO may be associated with further complications, such as CME requiring prolonged treatment [73]. Therefore, combined phacoemulsification with intraocular lens implantation at the time of SO tamponade may be considered a reasonable management option for selected phakic patients with RRD.

In conclusion, anterior segment complications associated with SO are common and multifactorial, with corneal endothelial damage and near-universal cataract progression driven by emulsification, anterior migration and tamponade duration. Early endothelial monitoring is therefore may be beneficial, along with consideration of combined cataract extraction at the time of SO tamponade in phakic eyes.

### Retinal layers in eyes with silicone oil

#### Eyes with silicone oil in situ

In eyes where SO remained in situ, OCT often revealed transient changes in inner retinal layer thickness compared to the contralateral eye – both in macula-on [12, 74] and -off RRD cases [75–77]. Several studies report that such variations are reversible, resolving after ROSO, and are not typically associated with changes in visual acuity [12, 76]. It is important to note that SO may affect the quality of OCT imaging [74], particularly of the RNFL [78], although some studies report that the reproducibility of foveal thickness measurements is not compromised [79].

#### Morphological macular changes

Vitrectomy for RRD repair using SO can result in CME, with a reported incidence ranging from 14% to 36% [51, 80]. In addition to the inflammatory response associated with SO, a mechanical mechanism has been proposed for CME development: an adhesive interaction between SO and the retinal surface that mimics a vitreomacular traction syndrome and typically resolves after ROSO [80]. Identified risk factors for CME following RRD surgery with SO include lower SO viscosity [51], the presence of posterior staphyloma [81], a higher preoperative grade of PVR [82], and longer SO tamponade duration [82, 83]. Management of SO-associated CME includes ROSO, topical corticosteroids and non-steroidal anti-inflammatory drugs, as well as intravitreal corticosteroids such as triamcinolone or dexamethasone; however, the vitreous half-life of intravitreal agents is reduced after PPV [72, 80–82]. Intravitreal anti–vascular endothelial growth factor therapy has also been shown to be effective in the management of CME, either before or after ROSO [84].

The formation of epiretinal membranes (ERM) after PPV for RRD is associated with macula-off, older age, a larger RRD extent, giant retinal tears, and the use of SO [85, 86]. The incidence of ERM following RRD varies from 4.6 to 70% [50, 85]. In RRD cases with SO, the prevalence of ERM was found to be between 12.3% and 70.6%, and strongly correlated with higher grades of PVR, longer duration of SO tamponade and photocoagulation energy [49, 86, 87]. In histopathological analyzes, macrophage cell counts were significantly greater in retro-oil ERM, compared to idiopathic or PVR membranes, which suggests an inflammatory mechanism for SO-associated ERM [88, 89]. The SO-associated ERM are also double-layered and have a spongy consistency, making surgical removal more difficult [89]. The use of triamcinolone during RRD surgery and ILM peeling before SO were associated with a lower incidence of ERM [49, 50].

#### Eyes with macula-off RRD

In macula-off RRD cases treated with SO, multiple studies reported significant thinning of the ganglion cell layer (GCL), inner plexiform layer (IPL), and outer plexiform layer (OPL) after ROSO, compared to eyes treated with gas [90, 91]. Another study found increased thickness in the inner nuclear layer (INL) and OPL but thinning in the outer nuclear layer (ONL) in SO-treated eyes, compared to the contralateral eye [92]. After macula-off RRD, outer retinal layers frequently undergo structural damage due to ischemia and apoptosis [93]. One consideration is that eyes in the SO group often undergo two surgeries (initial RRD repair and later SO removal), unlike the gas group [94]. Inflammatory cell infiltration – particularly macrophages – has been noted during tamponade and following ROSO, contributing to transient retinal thickening [74]. In addition, the thickening of the INL can be explained by the fact that Müller cells increase their activity after an aggression to the retina, such as RD: they grow and invaginate towards the subretinal space in prolonged detachments, which could affect the INL thickness [92, 95–97]. Several glaucoma studies have also reported an inverse relationship between RNFL thinning and INL thickening or microcystic changes, suggesting secondary remodelling of the inner nuclear layer in glaucoma [98, 99]. These findings suggest that both the detachment itself, glaucoma, and SO tamponade may contribute to retinal layer alterations, though their independent effects remain difficult to isolate, and will likely require prospective or controlled study designs to disentangle their independent contributions [100].

#### Eyes with macula-on RRD

Multiple studies have described thinning of both inner and outer retinal layers in eyes with macula-on RRD treated with SO, compared to either the contralateral eye [53], or gas-treated eyes [25, 94, 101], besides thinning of the outer retinal layers as well. [94] However, limitations exist. One study used pixel rather than micron units and omitted standard deviation reporting [25], and another had a relatively short follow-up (12 weeks). [101] Longer follow-up periods are essential for assessing retinal thickness changes, given that photoreceptor outer segment elongation after reattachment may normalize over several months. [102].

Several hypotheses have been proposed regarding the mechanisms of inner retinal damage due to SO, namely: (i) mechanical compression in the superior parafoveal area from SO contact during upright positioning;^91^ (ii) intraretinal migration of emulsified SO via ILM defects or macrophage infiltration [34, 94, 103, 104]; (iii) toxic effects of LMWC, which may activate microglia, trigger inflammation, and induce retinal cell death [90, 91, 94]; (iv) disruption of potassium homeostasis due to Müller cell dysfunction [21, 105]; (v) retinal dehydration caused by the hydrophobic nature of SO displacing the natural hydrophilic environment of the vitreous [76]; and (vi) SO-related changes in the choroid and retinal vascular plexus may further compromise perfusion [28, 101, 106].

The extent to which macular thinning is secondary to optic neuropathy remains debated. [25] Thinning of the ganglion cell complex has been observed in both macula-on [25, 27, 53] and -off eyes,^77^ regardless of glaucoma or OHT [27, 76, 77, 91, 92, 107]. However, some authors excluded patients with known glaucoma [27, 91, while others linked RNFL thinning to OHT [53].

In conclusion, studies evaluating changes in retinal layers following SO tamponade should ideally report the macular status (macula-on or -off), whether OCT measurements were obtained with SO in situ, and the nature of comparisons made, whether longitudinal within the same eye, *versus* gas tamponade, or *versus* the contralateral eye. Finally, establishing consistent correlations between structural alterations and visual acuity outcomes is critical to determine the true clinical relevance of these findings.

### Vascular changes associated with silicone oil use

#### Retinal circulation

In eyes with SORVL, whether the detachment was macula-on [28] or -off [106], a marked reduction in vessel density within the retinal superficial capillary plexus has been reported, when compared to the contralateral eye. In a comparative study of macula-on RRD eyes treated with SO *versus* gas, SO was associated with a greater reduction in both superficial and deep retinal blood flow. However, a trend toward recovery of retinal perfusion was observed over the first 6 weeks postoperatively [101]. It was hypothesized that strict postoperative prone positioning may lead to mechanical compression of the superficial capillary plexus by SO, causing local ischemia and ganglion cell apoptosis. Lou and co-authors assessed retinal oxygenation using retinal oximetry in patients who underwent 20G vitrectomy with SO. They found that SO retention beyond 9 months led to retinal arteriole narrowing. This has been hypothesized to reflect neuroretinal toxicity or to elevated intraretinal oxygen levels [108]. Earlier works by Effert and co-authors reported a prolonged arteriovenous passage time in SO-filled eyes, using laser scanning ophthalmoscopy, compared with contralateral eyes. This may suggest impaired retinal microcirculation, particularly in the early postoperative phase [109].

#### Choroidal circulation

Most studies report choroidal thinning following SO tamponade, regardless of diurnal variation or endolaser effects [95, 110]. A negative correlation between SO duration and subfoveal choroidal thickness has also been described, suggesting that prolonged SO exposure may contribute to choroidal microangiopathy or inflammation [111]. Macrophage infiltration in ocular tissues after SO exposure has been documented, possibly impairing foveal choroidal blood flow [110, 111]. Conversely, a separate short-term follow-up study found no significant difference in choroidal thickness between SO- and gas-treated eyes [101]. This may suggest that dynamic changes in choroidal blood flow – rather than static structural changes – might serve as a more sensitive indicator of SO-related choroidal alterations [101].

### Ocular hypertension and silicone oil-related glaucoma

The impact of vitrectomy on IOP is complex and varies depending on the tamponade used and patient-specific factors. Following vitrectomy without tamponade, the incidence of OHT ranges from 4.2% to 19.2% [112, 113]. This is thought to result from increased intraocular oxygen levels, which may promote oxidative stress and damage the trabecula, increasing resistance to aqueous outflow [112, 113]. Conversely, other studies report a trend toward IOP reduction after vitrectomy, possibly due to decreased episcleral venous pressure or reduced ciliary body perfusion from elevated oxygen tension [114].

When SO is used, OHT is more prevalent, with reported incidence between 11% and 56% [34, 57, 115]. There is no consensus on which preoperative factors reliably predict SO-induced glaucoma, although some studies suggest associations with preoperative OHT [57, 58, 116] and diabetes mellitus [116] particularly in PDR. However, these associations may not apply in cases of RRD [57, 117].

Mechanisms of SO-induced glaucoma include: (i) SO overfill, (ii) pupillary block, (iii) emulsification with infiltration of the trabecula, and (iv) migration of SO bubbles into the AC, where it may cause mechanical obstruction or trabecular toxicity [34, 41, 58]. Although viscosity does not appear to significantly affect IOP,^55^ higher IOP correlates strongly with the height of the emulsified SO meniscus in the AC, on ultrasound biomicroscopy [118]. Lower-viscosity SO has also been associated with earlier emulsification and higher glaucoma risk in some studies,^57,58^ though these findings contradict others discussed previously [5, 12, 54]. The presence of LMWC, impurities, or inflammatory mediators may further increase risk by promoting emulsification or trabeculitis [34].

Inflammation plays a prominent role in the pathogenesis of SO-induced OHT. Elevated IOP has been noted, albeit rarely, in immunocompromised patients (e.g., CMV retinitis, HIV), likely due to altered immune responses. [58] Liu and co-authors demonstrated increased levels of interleukin (IL)-6, IL-17, and tumor necrosis factor (TNF)-α in the aqueous humour of SO-filled eyes with glaucoma, mirroring the inflammatory profile seen in uveitic glaucoma [119]. Microbubbles in contact with the trabecular meshwork may trigger chronic low-grade inflammation [58, 120]. Additionally, SO may impede oxygen and nutrient diffusion between the vitreous and retina, possibly leading to RNFL damage [58, 108, 119].

Several studies suggest that longer SO tamponade duration correlates with higher risk of OHT [116], likely due to chronic trabecular endothelial damage, collagen remodelling, and increased emulsification over time [5, 34, 38]. Fortunately, if SO is removed before irreversible damage occurs, IOP typically normalizes, and glaucomatous optic neuropathy is rare [58, 117]. The recent decline in SO-induced glaucoma rates is likely due to shorter SO duration and the use of highly purified SO free of LMWC.

In summary, current understanding of SO-related OHT and glaucoma indicates a multifactorial pathogenesis involving mechanical, inflammatory, and oxidative mechanisms. However, the critical analysis of these studies could not be done without limitations because (i) the definition of OHT after vitrectomy varied among studies [57, 114], (ii) the need for treatment with one or more eye drops was not always mentioned or accounted for in a standardised manner, (iii) gonioscopy was not routinely performed to find emulsified SO drops in the angle [57], and (iv) the diagnosis of glaucoma or progression was hard to establish once visual fields were not always reliable in RRD patients. Taken together, the current evidence supporting a multifactorial pathogenesis of SO-related OHT and glaucoma is largely based on heterogeneous and predominantly retrospective data, highlighting the need for prospective or controlled studies to better define risk factors and causal relationships.

### Electrolyte balance and cytokines during silicone oil

Recent studies have investigated the so-called subsilicone oil fluid (SSOF) – the fluid trapped between the SO and the retinal surface – or the composition of SO itself after centrifugation. These studies reveal that SO is not biologically inert, as previously thought [121–123].

Using gas chromatography, researchers have found that intravitreal SO contains lipophilic compounds, such as cholesterol and fatty acids, which appear to accumulate over time [124]. Their concentration increases with the duration of SO tamponade and is higher in younger patients. This suggests that SO can absorb substances from ocular tissues or the bloodstream. However, it is still unclear whether this absorption is harmful to the retina [19, 124]. Notably, the patients enrolled in these studies were reported to have conditions like endophthalmitis, PVR, PDR, or ocular trauma (not RRD) which may influence the results [124].

The “potassium theory” proposes that SO disrupts Müller cells’ ability to buffer excess potassium ions into the vitreous cavity. This could result in ion and cytokine accumulation in the SSOF, potentially harming the retina. If SO acts as a physical barrier to these substances, a sudden chemical shift may occur after ROSO, possibly triggering retinal damage from a spike in extracellular potassium [17, 19, 24, 29]. However, evidence is not consistent. Some studies show normal potassium, sodium, and chloride levels in SSOF [29], even in patients with SORVL [18]. Others found low magnesium and elevated lactate dehydrogenase in SSOF. Magnesium deficiency is linked to *N*-methyl-*D*-aspartate receptor-mediated retinal toxicity, though these changes might also be due to RRD itself. [29].

SSOF contains elevated levels of inflammatory cytokines such as IL-6, IL-8, monocyte chemoattractant protein-1 (MCP-1), and vascular endothelial growth factor, particularly in eyes with chronic inflammatory conditions, such as PVR or PDR [121, 123, 125, 126]. In these diseases, it has been shown that the vitreous has a higher concentration in the same cytokines, compared with non-diabetic and no-PVR controls [122, 123]. Some studies found that IL-6 concentration in SSOF differ from those in aqueous humour or vitreous of eyes with macular holes and primary vitrectomy but are similar to levels of IL-6 in the vitreous of eyes with PVR [121.] However, comparisons made between different eyes or different disease groups, limits generalizability, underscoring the need for controlled studies comparing matched populations [121, 126]. IL-6 promotes glial and fibroblast proliferation and may have a dual role: contributing to inflammation but also protecting photoreceptors [121, 123, 126]. MCP-1 has been associated with fibrous proliferation [123]. Importantly, cytokine levels in SSOF do not appear to change significantly with longer SO duration, suggesting a persistent local concentration effect.

In contrast to the hypothesis that SO contributes to inflammation, some evidence suggests that SO may reduce intraocular inflammation. In diabetic tractional RD, SO was associated with lower levels of IL-6 and IL-8, and higher levels of IL-10, suggesting a potential anti-inflammatory effect [127]. However, most inflammatory cytokines in SSOF are not soluble in SO itself. Therefore, even if SO acts as a barrier, the SSOF may expose the retina to concentrated proinflammatory factors that could contribute to retinal damage [19].

To summarize, SO may alter the intraocular biochemical microenvironment by acting as a barrier and reservoir for electrolytes and cytokines within the SSOF, potentially exposing the retina to sustained ionic imbalance and inflammatory mediators, although the clinical relevance and directionality of these effects remain incompletely understood and likely disease dependent.

### Globe deposits

The toxic effect of SO can also be caused by the migration of SO into ocular tissues. Firstly, SO particles can cross the ILM and penetrate the retinal layers, as was observed in rabbit eyes injected with SO [63]. “Encrustation figures” were found in rabbit retina, and these structures are believed to be the product of retinol and cholesterol extraction from the retinal cells caused by the SO. Also, some alterations were seen in endogenous lipid-containing bubbles commonly present in RPE cells of the rabbit retina [63]. Again, these data show that SO is not an inert substance, since it can dissolve lipids and affect physiological functions in the long term [63, 128]. These experiments were repeated in an in vivo rabbit model, including a control group, and there was no evidence of SO toxicity at 6 months. [34] Two in vitro studies with different methodologies showed opposite conclusions about the cytotoxicity of LMWC for retinal cells, including human ARPE-19 cells [8].

In human patients, SO droplets have been found in the Schlemm’s canal during a gonioscopy-assisted procedure [129]. In enucleated globes with SO after vitrectomy, vacuoles (both free and incorporated by macrophages) of SO were found in all layers of the retina, in the choroid, and in the AC angle [120]. The distribution of SO was not associated with the presence of T and B lymphocytes [104, 120, but it was closely associated with retinectomy sites, subretinal SO, and localised inflammation, supporting the idea that SO causes an inflammatory reaction [120]. In vivo studies have confirmed these findings. In fact, using OCT in patients with PVR, hyperreflective droplets of SO were found 3 months after surgery with SO above the optic nerve and intraretinally (in cystoid spaces) [47]. It remains unclear whether ILM peeling, retinotomies, or a high IOP are significant factors that contribute to the intraretinal migration of SO, though [47, 130]. The emulsified SO that remains trapped in the ocular tissues may explain the irreversible nature of SORVL [29].

Overall, SO can migrate beyond the vitreous cavity into retinal and anterior segment tissues, where emulsified droplets and LMWC may induce local inflammatory and toxic effects, potentially explaining the irreversible nature of SORVL despite ROSO.

### Optic neuropathy

Regarding optic neuropathy, SO can migrate into intraocular tissues, including the optic nerve, where it may exert direct toxic effects (with or without OHT) [41, 103]. This entity – termed pseudo-Schnabel’s cavernous degeneration – resembles classic Schnabel atrophy, and it is characterized by SO droplets within the optic nerve surrounded by activated macrophages and chronic inflammation [41, 120]. One paper reviewed studies from 1983 to 2012, describing SO-associated optic neuropathy in enucleated eyes due to retrolaminar migration of SO [104]. Almost all eyes had developed secondary glaucoma, although only a minority of cases had optic disc cupping [104]. Vacuoles of SO were found in 14/74 enucleated globes due to secondary angle closure glaucoma or *atrophia bulbi* in the optic nerve [103]. Explanations for these findings are: (i) optic disc congenital anomalies, (ii) the degeneration of the optic nerve due to infiltration of SO bubbles associated with OHT, and (iii) migration of phagocytosed emulsified oil bubbles [104]. In the same review, SO migration into the cerebral ventricles were described, despite the finding not having any clinical consequences [104].

In conclusion, SO can migrate into the optic nerve and induce a distinct inflammatory-toxic optic neuropathy, often in association with OHT but not necessarily with typical glaucomatous cupping, highlighting a rare yet severe pathway of SO-related visual damage with incompletely understood mechanisms.

## Conclusions

**Fig. 3:**
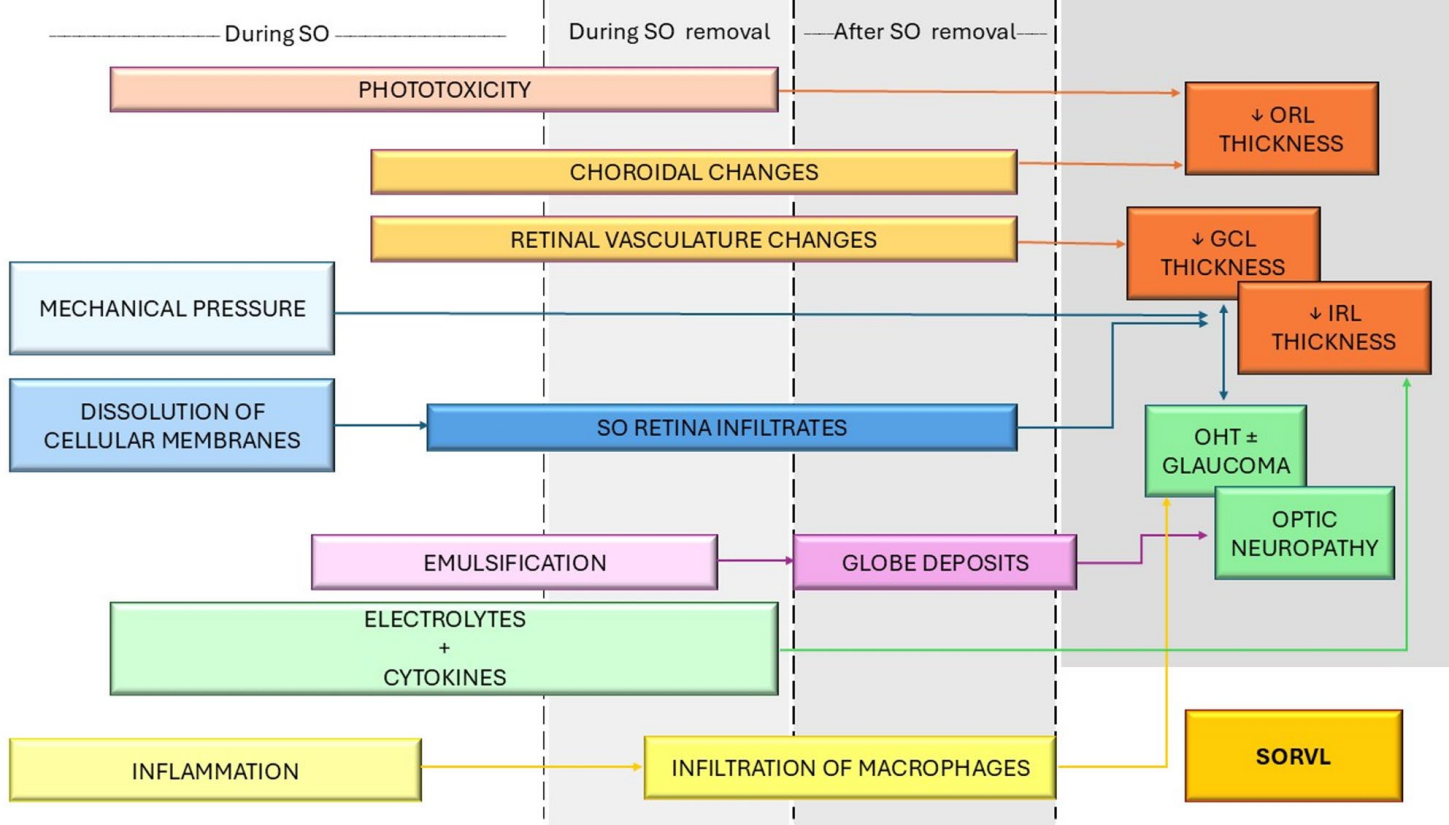
Silicone oil-associated structural changes and functional consequences (far right) and its mechanisms before, during and after silicone oil removal. GCL: ganglion cell layer; IRL: inner retinal layers; OHT: ocular hypertension; ORL: outer retinal layers; SO: silicone oil; SORVL: silicone oil-related visual loss

This narrative review highlights the multifactorial nature of SO complications and the need for more standardized clinical guidelines. Although SO remains a critical tool in managing complex RRD, its associated adverse effects, particularly SORVL, are often unpredictable and poorly understood. The underlying mechanisms likely involve a complex interplay of retinal toxicity, vascular compromise, and immune responses (Fig. 3). Preoperative individual factors and SO factors such as purity, viscosity, and duration of tamponade are clinically relevant but insufficiently studied, and current evidence lacks consistency in methodology and outcome reporting. Given the potential for irreversible damage, early detection protocols, and consensus on timing for SO removal are needed. Future research should focus on well-designed prospective and controlled studies, incorporating longitudinal clinical data, multimodal imaging, and molecular analyses, to clarify the pathophysiology and improve risk stratification. A multidisciplinary approach involving bioengineers, and basic scientists will be essential to refine SO use and guide the development of safer and more biocompatible vitreous substitutes.

## Supplementary Information

Below is the link to the electronic supplementary material.

Supplementary Material 1

## Data Availability

All data generated or analyzed during this study are included in this published article and its additional files.

## Declarations

### Generative AI and AI-assisted technologies in the writing process

During the preparation of this work the authors used ChatGPT to assist in checking grammar and enhancing clarity. After using this tool, the authors reviewed and edited the content as needed and took full responsibility for the content of the published article.

## Abbreviations

AC: Anterior chamber
CME: Cystoid macular edema
ERM: Epiretinal membrane
IOP: Intraocular pressure
GCL: Ganglion cell layer
IL: Interleukin
ILM: Internal limiting membrane
INL: Inner nuclear layer
IPL: Inner plexiform layer
LMWC: Low-molecular-weight SO components
MCP-1: Monocyte chemoattractant protein-1
OCT: Optical coherence tomography
OHT: Ocular hypertension
ONL: Outer nuclear layer
OPL: Outer plexiform layer
PDR: Proliferative diabetic retinopathy
PVR: Proliferative vitreoretinopathy
RNFL: Retinal nerve fiber layer
ROSO: Removal of silicone oil
RPE: Retinal pigment epithelial
RRD: Rhegmatogenous retinal detachments
SO: Silicone oil
SORVL: Silicone oil-related visual loss
SSOF: Subsilicone oil fluid
TNF: Tumor necrosis factor
